# Hip Joint Stresses Due to Cam-Type Femoroacetabular Impingement: A Systematic Review of Finite Element Simulations

**DOI:** 10.1371/journal.pone.0147813

**Published:** 2016-01-26

**Authors:** K. C. Geoffrey Ng, Mario Lamontagne, Michel R. Labrosse, Paul E. Beaulé

**Affiliations:** 1 Department of Mechanical Engineering, University of Ottawa, Ottawa, Ontario, Canada; 2 School of Human Kinetics, University of Ottawa, Ottawa, Ontario, Canada; 3 Division of Orthopaedic Surgery, University of Ottawa, Ottawa, Ontario, Canada; University of Umea, SWEDEN

## Abstract

**Background:**

The cam deformity causes the anterosuperior femoral head to obstruct with the acetabulum, resulting in femoroacetabular impingement (FAI) and elevated risks of early osteoarthritis. Several finite element models have simulated adverse loading conditions due to cam FAI, to better understand the relationship between mechanical stresses and cartilage degeneration. Our purpose was to conduct a systematic review and examine the previous finite element models and simulations that examined hip joint stresses due to cam FAI.

**Methods:**

The systematic review was conducted to identify those finite element studies of cam-type FAI. The review conformed to the Preferred Reporting Items for Systematic Reviews and Meta-Analyses guidelines and studies that reported hip joint contact pressures or stresses were included in the quantitative synthesis.

**Results:**

Nine articles studied FAI morphologies using finite element methods and were included in the qualitative synthesis. Four articles specifically examined contact pressures and stresses due to cam FAI and were included in the quantitative synthesis. The studies demonstrated that cam FAI resulted in substantially elevated contact pressures (median = 10.4 MPa, range = 8.5–12.2 MPa) and von Mises stresses (median 15.5 MPa, range = 15.0–16.0 MPa) at the acetabular cartilage; and elevated maximum-shear stress on the bone (median = 15.2 MPa, range = 14.3–16.0 MPa), in comparison with control hips, during large amplitudes of hip motions. Many studies implemented or adapted idealized, ball-and-cup, parametric models to predict stresses, along with homogeneous bone material properties and *in vivo* instrumented prostheses loading data.

**Conclusion:**

The formulation of a robust subject-specific FE model, to delineate the pathomechanisms of FAI, remains an ongoing challenge. The available literature provides clear insight into the estimated stresses due to the cam deformity and provides an assessment of its risks leading to early joint degeneration.

## Introduction

**Fig 1:**
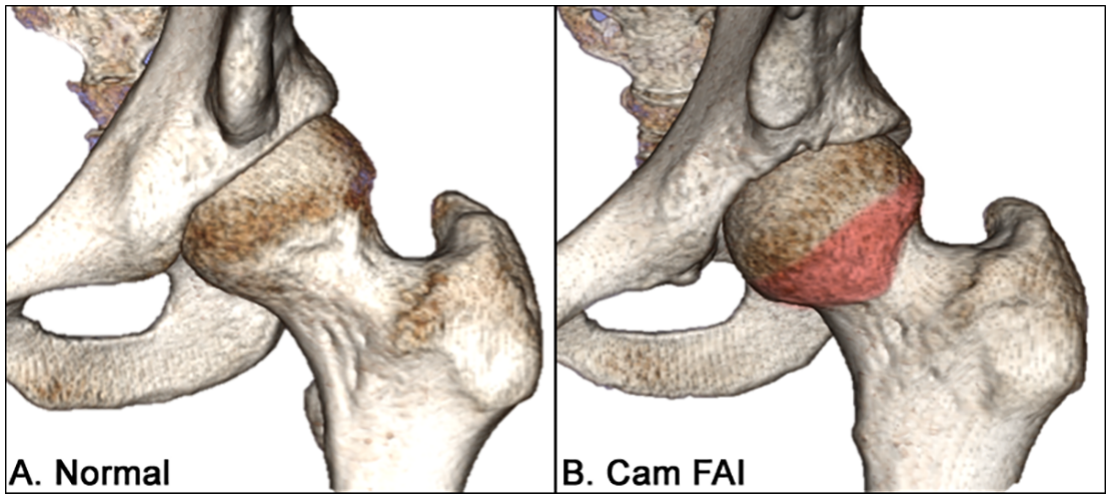
Comparison between a normal and a cam FAI hip. Three-dimensional models representing a healthy, normal left hip joint (A) and a left hip joint with severe cam-type femoroacetabular impingement (B), with the cam deformity highlighted in red.

The morphologies leading to mechanical femoroacetabular impingement (FAI) can be distinguished as either cam (enlarged femoral head deformity), pincer (acetabular over-coverage), or a combination of both [1, 2]. The cam-type deformity is characterized by a decreased head-neck offset [1, 3, 4], giving it a pronounced anterolateral bump with lack of concavity at the femoral head-neck junction [5, 6] (Fig 1). It has been attributed to adverse hip trauma and loading [1, 6, 7], significant athletic activity [7–9], and contact sports [10, 11], prior to skeletal maturation. Individuals with a larger cam deformity, as defined by higher alpha angles [12], ultimately leads to a greater risk of the anterosuperior femoral head obstructing with the acetabulum during combined motions of hip flexion, rotations [6, 8, 13–17], and squatting [15, 16, 18–20], resulting in early adult cartilage degeneration [1, 14, 21, 22].

FAI is rarely painful in its early stages, thus can go unrecognized for several years during its preliminary asymptomatic settling-in phase [23–26]. Early diagnosis and treatment of FAI is important to alleviate the risk of severe hip pain, irreversible cartilage damage, and osteoarthritis (OA). The difficulty with early diagnosis is that the deformities appear to look normal during its early stages of development [10], when there is an evident lack of focus in implementing additional visualization or diagnostic tools to assess the severity of the deformity leading to FAI.

Although diagnostic imaging (e.g. x-ray, computed tomography (CT), magnetic resonance imaging (MRI)) is the standard practice to confirm the presence of any hip deformity, it may be very difficult to determine if an individual will show any symptoms or indicate FAI, especially if dynamic hip motions are not performed. From previous motion analyses, symptomatic patients demonstrated constrained hip motions, such as during level walking [27–29] and squatting [17, 18]. Additional studies that involved finite element (FE) modelling and analysis examined resultant hip joint stresses due to cam FAI, providing a better picture of the pathomechanism. Many *in silico* simulations shared similar FE methods, however, posed various research questions that resulted in different observations and dependent variables. Moreover, while several studies implemented different mechanical stress analyses, it was unclear which were more applicable to assess adverse loading conditions in the hip joint due to cam FAI. In efforts to examine the effects of cam-type FAI on mechanical hip joint loading and to better understand the causal relationship between mechanical stimulus and cartilage degeneration, our purpose in this systematic review was to examine previous studies involving finite element analysis (FEA) that simulated hip joint loading due to cam-type FAI and determined hip joint stresses.

## Methods

The systematic review conformed to the Preferred Reporting Items for Systematic Reviews and Meta-Analyses (PRISMA) guidelines (S1 Appendix). The protocol started with a literature search, from three electronic databases: PubMed, Web of Science (Thomas Reuters), and Cochrane Library. The protocol subsequently consisted of a screening process, to further justify the pertinence and eligibility, and was completed on February 28, 2015.

### Identification

A general search was conducted in each of the three online databases using the terms “femoroacetabular impingement”, “femoro-acetabular impingement”, and “hip impingement”, with any of the terms to appear as a keyword or within a field of the article. Among the articles, the earliest was defined by Myers and associates (1999) [11], thus the time period for the literature search was limited from 1999 to 2015. This preliminary search resulted in 2559 combined articles from the three databases.

### Screening and Eligibility

The articles were imported into a citation management program (EndNote X4, Thomas Reuters, Philadelphia, PA, USA) where duplicates were removed. Among the remaining articles, a second search used the term “finite element” and further narrowed down the pertinent literature (n = 17). The abstracts of the remaining articles were then reviewed for eligibility and any study that did not examine a cam or pincer morphology was excluded. The included articles were reviewed and a qualitative synthesis compared each study’s methodology. A quantitative meta-analysis was conducted on the studies that specifically examined hip joint stresses due to cam FAI. Measureable dependent variable and stress parameters were thoroughly examined in each of the eligible articles, looking specifically for “von Mises stress” (or “maximum-distortion energy”), “maximum-shear stress” (or “Tresca stress”), or “contact pressure”. Studies that reported results with a common dependent variable were grouped together for the meta-analysis.

## Results

**Fig 2:**
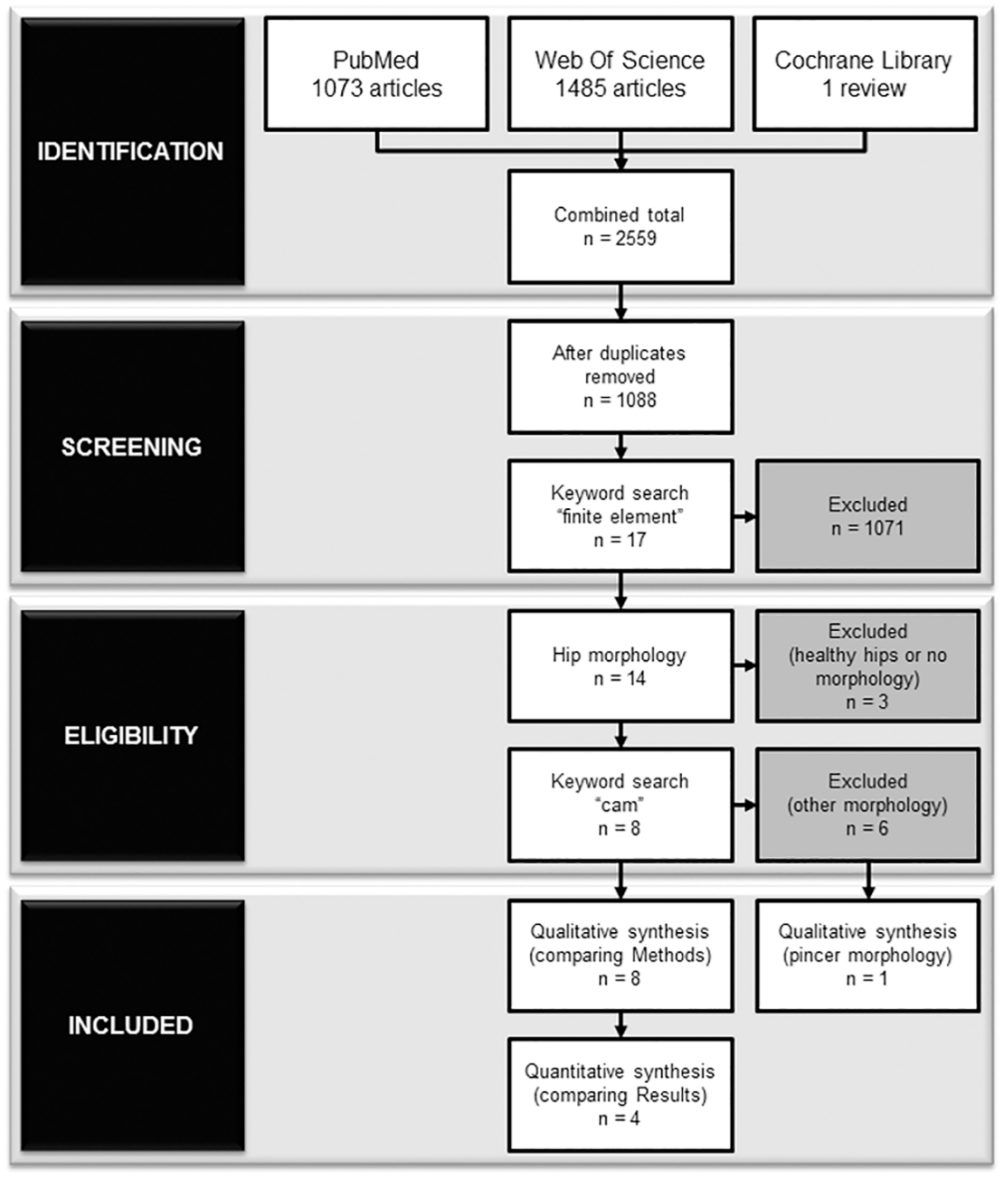
Flowchart of selection criteria. According to the PRISMA guidelines, the number of articles started with a total of 2559 combined articles from 3 databases (PubMed, Web of Science, and Cochrane Library). From those, a total of 9 and 4 articles were included in the qualitative and quantitative syntheses, respectively.

A total of 9 articles, in which a cam (8) or pincer (1) hip deformity was simulated using FEA, were deemed eligible and included in the qualitative synthesis. From those, a total of 4 articles examined hip joint contact pressures or stresses due to cam FAI (Fig 2).

### Preliminary Parametric Models

The first documented simulation that examined cam FAI was performed by Chegini and associates, focusing on impingement and dysplasia during sitting and walking [16]. The simulations comprised of a spherical, ball-and-cup FE model that was parameterized to various alpha and lateral centre-edge (CE) angles, according to the severity of cam FAI (higher alpha angle), pincer FAI (higher CE angle), or dysplasia (lower CE angle). The advantage with using an idealized, parametric model was that the deformities were easily defined and simulated at every 10-degree increments for alpha and centre-edge angles (alpha angle = 40 to 80°; CE angle = 0 to 40°). The study took a single patient and considered two loading scenarios—walking and stand-to-sit. Hip contact loads were taken from *in vivo* instrumented prostheses data and applied through the femoral head [30]. No specifications were given on patient details and dimensions of the final model. Peak contact pressures and von Mises stresses were found in the acetabular cartilage for both activities. During walking, no adverse stresses were noticed with increasing alpha angles. This justified that peak stresses would be more prominent at higher dynamic motions (i.e. higher stresses during squatting motions, as opposed to walking). For the stand-to-sit activity, higher alpha and CE angles resulted in higher contact pressures and stresses.

Chegini and associates’ study only considered the oblique-axial plane when varying alpha angles and did not consider radial planes. Moreover, the cam and pincer deformities were limited to an alpha angle and CE angle of 80° and 40°, respectively, thus a more severe cam or pincer deformity was not observed. The cam-only deformity cases showed peak contact pressures that varied from 3.67 to 12.84 MPa (alpha angle = 60 to 80°, CE angle = 20 to 30°); and von Mises stresses from 9.7 to 27.2 MPa (alpha angle = 60 to 80°, CE angle = 20 to 30°), situated at the anterosuperior cartilage. Their mixed impingement cases demonstrated slightly higher peak contact pressures from 10.52 to 16.51 MPa (alpha angle = 60 to 80°, CE angle = 40°); and much higher von Mises stresses from 30 to 37 MPa (alpha angle = 60 to 80°, CE angle = 40°), situated at the anterosuperior cartilage and labrum. It was uncertain if the acetabulum or innominate bone structure was included. The magnitudes of the von Mises stresses for the cam FAI models were provided, however, it was not explicitly reported for the control models. Only cartilage stresses and contact pressures were reported and no results pertaining to the acetabulum, pelvis, or femoral head were featured.

As a follow-up to Chegini and associates’ study, Arbabi and associates further examined penetration depths in the acetabular cartilage and labrum, indicating very high curvilinear and radial penetration due to the idealized geometries [31]. However, no specific information about the resultant von Mises stresses was provided. Although an idealized model was implemented, Chegini and associates contributed an exploratory study in the early phases of FAI research, which emerged as a preliminary benchmark.

### Subject-Specific Bone Models

The next set of simulations involving FEA attempted to improve the subject-specificity of the models. In an earlier work by Ng and associates, two patients with severe cam FAI were matched with two healthy control participants [19]. Each participant’s geometric model was segmented from subject-specific CT data and supplemented with subject-specific, intersegmental hip joint reaction forces. The manually segmented models provided a more realistic representation of the cam deformity, demonstrating the adverse loading conditions in the hip joint during standing and squatting. Elevated stresses were located on the anterosuperior bone surface, beneath the acetabular cartilage, during squatting for patients with severe cam FAI (15.2 ± 1.8 MPa), in comparison with healthy control participants (4.5 ± 0.1 MPa). This study provided a modelling perspective of cam FAI and integrated more subject-specific data to further understand the pathomechanism with mechanical stimuli corresponding to the known areas of acetabular cartilage damage. With elevated stresses on the bone surface (as opposed to direct loading on the cartilage), it emphasized the need to further understand the morphology and determine if joint degeneration may be due to the indirect changes in the subchondral bone. Although the inclusion of a labrum in FEA remains elusive [32], it would be beneficial in future studies to understand the residual physiological effect of the labral seal for this pathological hip condition [33–35]. In addition, the hip joint reaction forces provided valuable approximations of net forces and moments, but were still underestimations of *in vivo* contact forces. Future initiatives to include individual muscles and hip contact forces would better represent the physiological reactions and resultant hip contact stresses.

In contrast, Jorge and associates developed their subject-specific bone models from radial MRI, while cartilage and labrum were approximated using computer-aided design software [36]. Only one FAI patient model was developed (male, age = 27 years, alpha angle = 98°), matched with one healthy control model (female, age = 50 years, alpha angle = 48°). All soft tissues were considered linear elastic and isotropic, and the bones were assumed rigid. A compression load was applied to the femur on the acetabular cavity as well as flexural movements and internal rotations. Loading data from *in vivo* instrumented prostheses database were used in the simulations, using an arbitrary weight not specific to either participant. Jorge and associates also found an elevated peak contact pressure and von Mises stress in the anterosuperior cartilage (11.6 and 14.4 MPa, respectively) and labrum (16.4 and 14.7 MPa, respectively), during hip flexion for their FAI model, however, observed a substantially higher peak contact pressure (20.6 MPa) and von Mises stress (28.2 MPa) during partial and full internal rotation, respectively. The von Mises stress magnitudes for the control model was not explicitly reported. However, their study was limited by the sample size and poor matching criteria—one young male, with a severe cam deformity, matched with one healthy older female. This single comparison exhibited substantial differences, but may not have explained the differences or variations among the FAI and the control populations.

### Effects of Surgery

Alternatively, a few studies recently implemented FE models to examine the effect of surgical osteochondroplasty, specifically looking at the influence of resection depths with fracture loading and risks due to adverse loading [37, 38]. Alonso-Rasagado and associates developed a single FE hip model from CT data of a typical cam-type hip to predict stresses in the femoral head-neck junction after open surgical resection [37]. Based on this single hip model, bone resections were parameterized and performed virtually to incremental resection depths, instead of incorporating real post-operative CT data and hip joint loadings. No information was provided about the single femur model (e.g. sex, age, other morphologies). It was concluded that higher amplitudes of hip motion (e.g. knee bend and stairs descent) yielded the highest stresses when resection depth was beyond 10 mm. However, the authors used *in vivo* instrumented prostheses data (taken from an older population) which led them to reduce the elastic moduli of the bone models. This lacked a level of patient-specificity to represent the correct amount of bone resection for a younger population with cam FAI, although still suggesting a relative limit for resection depth.

Rothenfluh and associates used a general 3D femur model taken from a public anatomy database to simulate resection geometry on fracture risks [38]. Using *in vivo* data from instrumented prostheses data for stumbling and walking, they concluded that a resection should be limited to 20% depth and 35% length of the femoral neck. The group acknowledged that large inter-patient variations in bone quality, stature, and anatomy can occur; and, as a consequence, suggested that subject-specific models would greatly improve fracture risk predictions.

Liechti and associates expanded the early parametric hip models by Chegini and associates [16], to further examine pincer FAI and influences of contemporary surgical interventions on stress distributions in protrusio hips [39]. Material properties and pre-processing conditions were similar to the previous simulations [16, 31]. Hip joint loading data were again taken from *in vivo* instrumented prostheses data, for walking and stand-to-sit motions, and applied to several parameterized hip models (e.g. normal, dysplasia, protrusio) and surgical intervention methods (e.g. rim trimming and acetabular reorientation). No other models or components were considered, other than the cartilage and labrum components. The protrusio hip resulted in elevated contact pressures in the medial acetabular cartilage (1.62 MPa, 24% higher than their normal hip) and substantially higher von Mises stresses in the medial aspect of the posteroinferior acetabulum (54% higher than their normal hip). Acetabular reorientation decreased peak contact pressures, while additional rim trimming substantially reduced peak stresses. The authors noted that subject-specificity was not considered or addressed, as their models represented averaged geometries based on empirical, morphological data and not representative of a “larger spectrum of anatomy” [39].

### Cartilage Behaviours

A more recent FE study by Hellwig and associates adapted Chegini and associates’ parametric hip model [16] to examine cartilage stresses due to cam FAI [40]. Only two conditions were parameterized that compared a hip with cam FAI (alpha angle = 74°) and a healthy control hip (alpha angle = 40°). The cartilage component was modelled as a poroelastic, orthotropic material to characterize biphasic properties. Similar to the previous parametric studies [16, 31, 39], the activities of walking and stand-to-sit were simulated. As result, peak contact pressures for the normal hip were located in the superior cartilage during walking (2.87 MPa) and in the posteromedial cartilage during stand-to-sit (3.58 MPa). Peak pore pressure was noticeably different between the control model (0.42 MPa, in the posterior cartilage) and the FAI model (3.76 MPa, in the anterosuperior cartilage). No other bone component was considered in the analysis. The study implemented *in vivo* contact loads from the instrumented prostheses database [30], neglecting the subject-specificity of hip joint loading due to cam FAI. The study was also limited by a low sample size—one FAI condition matched with one control. Moreover, the authors noted that their 3D geometries were simplified and optimized for convergence, which may overlook the subject-specificity of inter-individual anatomical characteristics and material properties [40].

### Development of the Cam Deformity

A recent study by Roels and associates investigated mechanical factors leading to the development of a cam-type deformity [7]. A single FE model of the proximal femur was reconstructed from CT data (age = 12 years, left leg), parameterized with 3 different growth plate shapes, and simulated under 4 activities (normal walking, internal rotation, external rotation, hip flexion) using loading data from *in vivo* instrumented prostheses data. They implemented an osteogenic index to look at changes to the epiphyseal plate and followed up with their previous findings on young athletes undergoing skeletal maturation [8]. As a result, Roels and associates observed larger epiphyseal extensions during external rotation and hip flexion, with elevated osteogenic indexes localized where the cam deformity would likely develop. Unlike the previous models of cam FAI, they modelled the growth plate with a constant elastic modulus and considered heterogeneous bone material properties for the femur, taken from CT data, to better represent the varying densities.

In contrast with the other FE studies, Roels and associates’ intention was not to examine hip joint stresses, but rather to look at the development of the cam deformity and its association with various activities and loading parameters. Thus, their study and modelling parameters were reviewed and included in the qualitative synthesis to thoroughly examine pre-processing FE methods; however, since their study posed a different research question, their results were not included in the quantitative synthesis.

### Meta-Analysis

**Fig 3:**
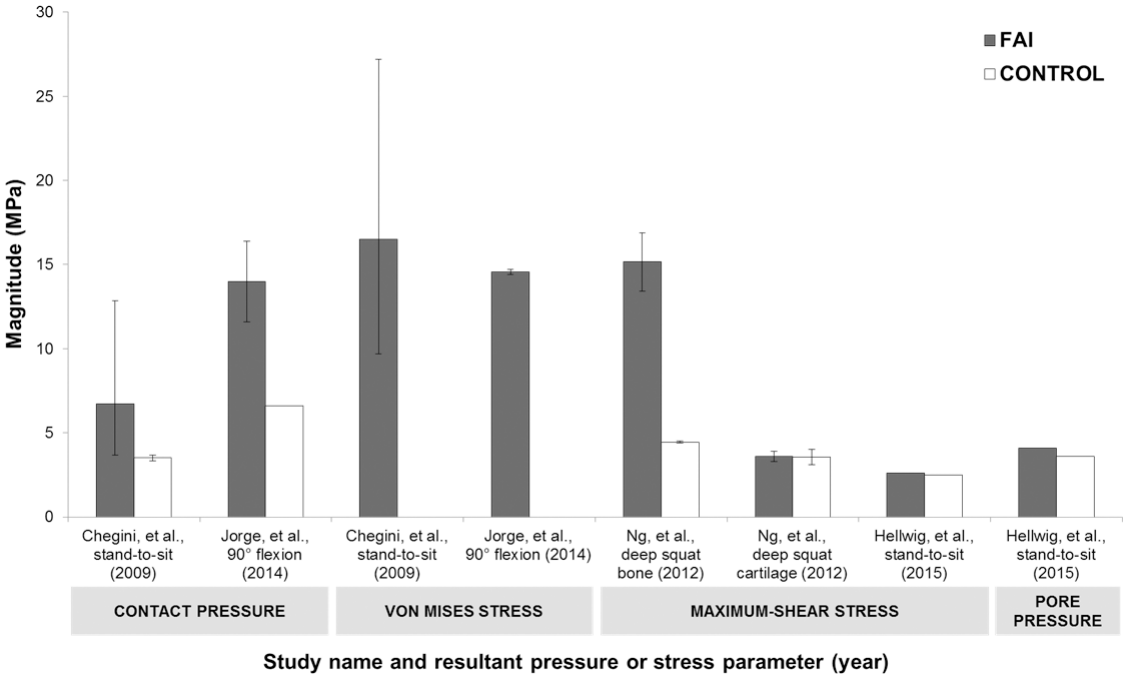
Summary of previous studies’ peak hip joint contact pressures and stresses. Peak contact pressure or stress on the acetabular cartilage or bone, during a deep hip flexion task for each study’s cam FAI (grey) and control group (white), reporting the averaged peak magnitude and maximum and minimum range. (The von Mises stresses for the control groups were not explicitly reported in Chegini, et al. 2009 and Jorge, et al. 2014, therefore, were intentionally omitted).

Table 1 lists the previous FE studies in literature that examined FAI—summarizing the study details, participant details, loading details, and results. Among the screened studies that had comparable quantifiable results, the most common dependent variables was an acetabular cartilage contact pressure or stress parameter during an activity that required larger amplitudes of hip motions (stand-to-sit, maximum squat depth, or deep hip flexion). Two studies reported peak contact pressures and peak von Mises stresses in the acetabular cartilage due to cam FAI, indicating a median contact pressure of 10.4 MPa (range = 8.5–12.2 MPa) and median von Mises stress of 15.5 MPa (range = 15.0–16.0 MPa) [16, 36], while two studies reported maximum-shear stresses, indicating a much lower median of 3.10 MPa (range = 2.84–3.35 MPa) [19, 40]. Only one of the studies examined stresses in the bone underneath the acetabular cartilage and indicated maximum shear-stresses of 15.2 MPa (range = 14.3–16.0 MPa) [19] and one study indicated a peak pore pressure of 4.10 MPa [40]. A comparison of all reported peak stresses for each study’s FAI and control groups can be seen in Fig 3.

**Table 1:** Previous studies on cam FAI that implemented finite element methods, summarizing the study detail, modelling and simulation methods, and results of the cam FAI group.

Study Details			Participant Details		Loading Details		Results	
Scope	Author (year)	Purpose	Sample Size	Model	Activities	Methods	Peak Stress Magnitude	Peak Stress Location
**Stresses due to cam deformity**	Chegini, et al. [16] (2009)	Contact pressure and stress in cam and pincer FAI, dysplasia	n = 1 (25 conditions, parameterized for alpha and center-edge angles)	Spherical, ball-and-cup model; uniform cortical shell; with linear-elastic, isotropic bone and cartilage	Walking and stand-to-sit	Percentage of bodyweight load, from *in vivo* instrumented prostheses data	Contact pressures from 3.67 to 12.84 MPa and von Mises stresses from 9.70 to 27.20 MPa, during stand-to-sit	Anterosuperior cartilage and labrum, during stand-to-sit
	Ng, et al. [19] (2012)	Stresses on cartilage and bone layer due to cam FAI	n = 4 (2 cam males; 29, 44 years; alpha angle = 74, 84°; matched with 2 control males; 36, 54 years; alpha angle = 41, 45°)	Subject-specific hip joint geometry, from CT data; variable cartilage thickness; with orthotropic bone and isotropic cartilage	Standing and squatting	Subject-specific intersegmental reaction forces from inverse dynamics	Maximum-shear stress in cartilage from 3.3 to 3.9 MPa and in bone from 13.4 to 16.9 MPa, during squatting	Anterosuperior quadrant of acetabulum, during squatting
	Jorge, et al. [36] (2014)	Contact pressure and stress on cartilage due to cam FAI	n = 2 (1 cam male, 27 years, alpha angle = 98°; matched with 1 control female, 48 years, alpha angle = 48°)	Subject-specific geometry, from MRI; no information on bone model or materials; linear-elastic, isotropic soft tissues	Joint compression with full flexion and internal rotation	Percentage of bodyweight load, from *in vivo* instrumented prostheses data	Contact pressures from 11.6 to 16.4 MPa and von Mises stresses from 14.4 to 14.7 MPa, during flexion	Anterosuperior cartilage and labrum, during flexion
	Hellwig, et al. [40] (2015)	Cartilage behaviour due to cam FAI	n = 2 (1 cam, alpha angle = 74°; matched with 1 control, alpha angle = 40°)	Spherical, ball-and-cup model; uniform cortical shell; linear elastic, isotropic bone with poroelastic, orthotropic cartilage	Walking and stand-to-sit	Percentage of bodyweight load, from *in vivo* instrumented prostheses data	Contact pressure of 4.09 MPa and Tresca stress of 2.59 MPa, during stand-to-sit	Posteromedial cartilage, during stand-to-sit
**Penetration Depth**	Arbabi, et al. [31] (2010)	Penetration depth and stresses in labrum	n = 1 (25 conditions, parameterized for alpha and center-edge angles)	Spherical, ball-and-cup model; uniform cortical shell; with linear elastic, isotropic bone and cartilage	Stand-to-sit	Percentage of bodyweight load, from *in vivo* instrumented prostheses data	High curvilinear and very high radial penetration; no details on peak stress magnitude	Anterolateral labrum
**Development of cam deformity**	Roels, et al. [7] (2014)	Loading on epiphyseal growth plate	n = 1 (male, 12 years; parameterized for flat and convex growth plate shapes)	Subject-specific femur geometry, from CT data; with subject-specific bone material properties, based on empirical formula	Walking, internal rotation, external rotation, deep flexion	Percentage of bodyweight load, from *in vivo* instrumented prostheses data	Osteogenic index of 0.7 MPa, during external rotation; noticeable increase in osteogenic index, during external rotation and flexion	Superolateral side of growth plate, during external rotation
**Effects of surgery**	Alonso-Rasagado, et al. [37] (2012)	Stresses on femoral head-neck after cam resection	n = 1 (6 conditions, parameterized for various resection depths)	Subject-specific femur geometry, from CT data; with elastic-plastic, isotropic bone	Single and double leg stance, walking, stairs descent, knee bend	Percentage of bodyweight load, from *in vivo* instrumented prostheses data	von Mises stresses of 16 to 17.5 MPa, at resection depth > 10 mm, during knee bend	Superolateral femoral neck, with resection depth > 10 mm, during knee bend
	Rothenfluh, et al. [38] (2012)	Fracture loads after cam resection	n = 1 (3 conditions, parameterized for various resection depths)	Subject-specific femur geometry, from anatomy database; with linear elastic, isotropic bone	Stumbling, fast walking, normal walking	Percentage of bodyweight load, from *in vivo* instrumented prostheses data	Critical fracture load = 4150 N, at 30% resection (28 mm length, 39 mm width	Location of fracture at inferomedial femoral neck
**Stresses due to pincer deformity**	Liechti, et al. [39] (2014)	Stresses due to pincer FAI	n = 1 (6 conditions, parameterized center-edge angles for various acetabular shapes)	Spherical, ball-and-cup model; uniform cortical shell; with linear elastic, isotropic bone and cartilage	Walking and stand-to-sit	Percentage of bodyweight load, from *in vivo* instrumented prostheses data	Contact pressure of 1.62 MPa, for protrusio hip during stand-to-sit	Posteromedial cartilage (5.1 mm from medial margin, with respect to acetabular arc), for protrusio hip during stand-to-sit

## Discussion

### Model Predictions

The previous generation of *in silico* FE studies provided clear objectives in examining contact pressures and stresses due to cam FAI, with each study’s parameters justifying their initial research questions. The simulations provided a concrete understanding of pathological joint loading and showed that the cam morphology led to substantially elevated stresses in the acetabulum at a higher range of hip motion. The different studies implemented various methods to characterize hip joint contact mechanics, using different parameters to measure contact stresses, thus making a direct comparison of dependent variables slightly more difficult. The extracted data indicated that contact pressure and von Mises stress were higher in the acetabular cartilage [16, 36], in comparison with maximum-shear stress and pore pressure [19, 40], in cam FAI models.

As with any *in silico* study, there are many limitations associated with FE methods and its resultant predictions. As noted by Viceconti and associates, sensitivity and validity are still ongoing challenges to be addressed in FEA, especially when the study involves complex multicomponent, musculoskeletal systems [41]. They further outlined that simulations should implement a well-defined and assessed model with correctly identified and verified input parameters, in efforts to ensure accurate and representative predictions. Many of the previous FE studies of cam FAI featured convergence analyses to address concerns of meshing sensitivities, but their conclusions were still cautious. The FE predictions were often validated against case controlled hip joint simulations, involving other similar FE hip models, or validated against previous clinical observations.

A comparison with clinical observations can partially justify the validity of predictive models. As many of the FE simulations demonstrated, the cam morphology can lead to acetabular cartilage damage predominantly in the anterosuperior region [21, 22, 42–44]. Beck and associates observed 26 surgically dislocated hips and noted that the greatest depth of the cartilage lesions were located in the anterosuperior quadrant [22]. Similarly, Beaulé and associates observed 23 hips and noted cartilage lesions combined with labral tears, also located in the anterosuperior clock-face, at the time of surgery [43]. In addition, another study by Beaulé and associates reported severe acetabular cartilage damage in the anterosuperior region and further correlated the cartilage damage with elevated alpha angles [42]; whereas Clohisy and associates observed slightly higher articular cartilage abnormalities in the superolateral region, rather than the anterior periphery [44].

### Hip Joint Modelling

Comprehensive, subject-specific reconstructions were often avoided, perhaps due to the time-consuming efforts and complexities of imaging, geometric, and loading parameters, which ultimately resulted in lower inter-subject variability [41]. In most cases, FE simulations of the hip considered mainly bone models and the articulation components [16, 19, 31, 32, 45–47]. As a future improvement to the subject-specific material characteristics, FE hip joint models may be constructed from segmented bone and soft tissue geometries obtained from subject-specific CT and MRI data, using image segmentation software. It will be imperative to examine soft tissues landmarks around the hip joint. The inclusion of the labrum in hip joint modelling will also be crucial to understand the nature of the seal in pathological hip deformities [33–35]. The labrum can be reconstructed around the periphery of the acetabulum, creating a seal around the femoral head. To ensure that the articulation components as obtained from MRI are in accordance with the CT based bone models, the bone landmarks in each set of imaging data must be registered [48, 49].

Many studies of hip joint biomechanics have been simplified into 2D planar [46, 50] or idealized into a ball-and-socket analyses, which was deemed by some researchers as appropriate for preliminary approximations [51]. Knowing that the femoral head conforms to a conchoid shape rather than a perfect sphere [52], it would now be important to incorporate subject-specific contour data of the femoral head to accurately estimate the cartilage stresses and contact areas. Similar to the approach of CT-based navigation for preoperative planning, 3D FE models are now reconstructed using imaging data following a subject-specific approach [53]. Since the segmentation process is time-consuming, many studies implemented semi-automated segmentation methods to extract objects of interests from CT and MRI data [54–56]. The clarity and the sensitivity of the images tend to vary from scan to scan, often requiring more manual segmentation methods to ensure a higher level of confidence.

The selection of the material properties for FE modelling and simulations often depends on the physiological application and mechanical assembly of the models. Nonlinear effects of soft tissues can be approximated with hyperelastic [32] or poroelastic material properties [33, 57] to estimate responses. As for the bone geometries, some studies opted to apply an elastic modulus based on an empirical formula derived from the apparent density of bone [58–61]. A common limitation that all the previous FE studies on cam FAI had was that bone was modelled as a homogeneous material. It was argued in many studies that linear elastic models would be sufficient for quasi-static loading frequencies, and therefore two separate, linear elastic, isotropic entities to represent the cortical shell and internal trabecular structure were often implemented [16, 31, 39, 46, 47, 61]. However, knowing that bone is heterogeneous, the material properties would react differently in various locations. At minimum, since bone is macroscopically composed of cortical and trabecular bone with varying bone densities, there is a need to consider varying elastic moduli throughout its composition according to Hounsfield units from quantitative CT data.

In Anderson and associates’ FEA of the subject-specific hip joint (2008), bone was modelled as a hyperelastic, isotropic material (with tetrahedral elements), whereas cartilage was modelled as a neo-Hookean material (using brick elements) [45]. As a follow-up study, Anderson and associates (2010) used the same biomechanical approach to examine different modelling parameters—altering the femur’s and cartilage’s material models to examine their effects on stress predictions [32]. Their models neglected the trabecular bone component, arguing that a cortical shell was sufficient to demonstrate joint reactions [32, 45]. For the purpose of predicting the mechanical stimuli and areas of bone formation in a subject-specific fashion, it may be not be sensible to disregard the trabecular bone component when simulating FAI, as it provides inherent stability and the foundation of the bone remodelling matrix.

Anderson and associates’ latter study was meant as a comparative study to delineate the possible outcomes from various methods. Their parametric models demonstrated that spherical and conchoidal femoral head models, together with a smooth cartilage, distributed the stresses more evenly and underestimated stresses, in comparison with a subject-specific geometry. Furthermore, a constant cartilage thickness approach would be less realistic for pathologic hip deformities. It was not suggested which of their parametric hip models was the most correct; instead, it was described what should be the expectation in terms of stress patterns given specific input parameters. This further confirms that idealized models cannot adequately assess stress predictions and reiterates the need for subject-specific geometric models. In addition, Harris and associates reconstructed statistical shape models of hips with and without the cam deformity, from subject-specific CT data [62]. Although their intention was not FEA modelling, the statistical shape models compared the cam morphology with control femurs, emphasizing the need for subject-specific geometries. There was a noticeable difference between the two groups at the anterolateral head-neck junction, corresponding with the locations of cam deformity.

### Motion and Loading with Cam FAI

To further the understanding of subject-specific hip joint stresses, the joint loading should be specific and integrated with the associated hip joint model (i.e. subject-specific joint loading should not be approximated from instrumented prostheses data, if possible). Motion analysis can evaluate 3D kinematics and kinetics, during various activities of daily living. Since the structure and range of hip motion is vital to locomotion, standing upright, and performing many daily activities, it is important to determine how a deformity potentially impacts hip biomechanics. In a level-walking study by Kennedy and associates [27], it was found that walking biomechanics of cam FAI demonstrated marginal kinetic differences, when compared with a control group, but showed constrained abduction in the frontal plane. In contrast, Hunt and associates found lower hip extension, adduction, and internal rotation during the stance phase [63]. Lamontagne and associates further showed significant differences in pelvic motion between cam FAI and control subjects for a deep squat motion, where participants performed a maximum dynamic squat [17]. Their cam FAI group was unable to squat as low (41.5 ± 12.5%, as a percentage of leg height) compared with the control group (32.3 ± 6.8%, *p* = 0.037), suggesting that the maximal squat depth may be feasible as a diagnostic test [17]. This further motivates FE studies of cam FAI to involve larger amplitudes of hip motions.

In a follow-up study, post-operative patients (8 to 32 months after open surgical dislocation) were able to squat significantly lower (33.2 ± 10.3%), compared with their pre-operative performance (36.9 ± 12.0%, *p* = 0.027) [15]. However, these patients were unable to return to their pre-operative walking performance [29]. Contrarily, Rylander and associates found significant improvements in level-walking, confirming positive improvements for post-operative patients [28].

From 3D kinematics and kinetics data, net hip joint forces and moments can be calculated from inverse dynamics. These forces and moments represent intersegmental reaction loads, but the approach excludes individual muscle contributions, co-contractions, and other soft tissue loading (i.e. net passive moments). Nevertheless, the approach is still commonly used to estimate net joint moments and forces. To better understand the effect of cam FAI on the internal mechanical loading, hip contact forces are required and necessitate muscle and soft tissue loading contributions. *In vivo* contact force measurements are possible with instrumented prostheses [64–67]. The data received from instrumented prostheses contributed to numerous studies and publications, towards the better understanding of joint contact loading, and leading to a renowned online database. These studies were often limited by sample size and population, as most of the patients implanted with instrumented prostheses represented a specific age group and disease process (i.e. older population with severe arthritis, in contrast with a younger, athletic FAI population). Overall, this invasive method raises numerous technical and ethical concerns.

The approach followed by computational musculoskeletal models appears to be more general and versatile to estimate joint loadings, in efforts to include muscle contributions [68, 69]. The EMG-driven musculoskeletal models can implement a forward dynamics analysis to estimate muscle forces, from muscular activities [69–71], showing a better sensitivity to the subject-specific muscle activation [72]. The inclusion of muscle and contact forces obtained from such models may yield more complete and accurate loading conditions for FE analyses compared to what is currently available in the literature. To our knowledge, no FE study has implemented subject-specific muscle and hip joint contact forces, to examine resultant contact stresses.

### Asymptomatic Population

There has been growing interest to understand why many individuals with the cam deformity do not develop early symptoms of FAI [73–75]. Asymptomatic individuals are characterized by a cam-type deformity; however, do not demonstrate FAI (i.e. individuals with the cam deformity but do not demonstrate any impingement, clinical signs, symptoms, or pain). Although several studies examined the asymptomatic population, measuring additional anatomical parameters from radiographic [73, 75, 76], CT [20, 25, 77], or MRI [78–80] data; there are currently no FE studies that indicate hip joint stresses in the asymptomatic population. The health risk with the asymptomatic cam deformity is that it can remain undetected even though it predisposes to early joint degeneration. Ultimately, a closer examination of additional anatomical parameters, hip motion, joint loading and stresses, and correlation with resultant bone mineral density, might shed some light into possible clinical associations, and could greatly contribute to the to the understanding of the pathomechanisms at play in cam FAI.

## Conclusion

There is an evident trend to implement FEA toward the study of cam-type FAI. It was apparent that the previous FE studies were limited by low sample sizes and, at times, incorrectly matched groups, which perhaps indicate the rigorous and time-consuming efforts required to manually segment imaging data. Several of the previous studies implemented or adapted idealized, ball-and-cup, parametric models to predict hip joint stresses, in addition to homogeneous material properties and *in vivo* instrumented prostheses loading data. Although simplified for convergence, the parametric models in combination with *in vivo* hip contact loads measured from an older population may not be adequate to satisfy broader subject-specificity requirements. One of the biggest gaps in literature, and one of the ongoing challenges, is the formulation of a robust subject-specific FE model—one that will consider subject-specific parameters, hip joint loading, geometric models—that can predict the adverse loading conditions in the symptomatic and asymptomatic populations, ultimately delineating the pathomechanisms of FAI.

Moving forward, although there is strong suggestion from clinical observations that the presence of cam FAI presents a substantial risk of developing early hip OA, there are still large gaps in literature that cannot yet support such causality or account for different paths between the symptomatic and asymptomatic populations in the face of apparently similar mechanical effects due to the deformity. The data to accurately model, simulate, and understand the morphologies associated with FAI are still growing. Furthermore, very few studies incorporated subject-specific models to simulate biomechanical loading scenarios with the intention to address FAI. To better understand the pathomechanisms of cam FAI, one will have to answer the question: *what are the effects of cam FAI on mechanical hip joint loading*? Currently, although the results are not quite robust yet to reflect actual *in vivo* loading, as there is still room for improvement in terms of hip joint modelling, the available literature provides some insight into the estimated stresses due to the cam morphology; in turn this stress estimation may provide an assessment of the risk of early joint degeneration.

## Supporting Information

S1 Appendix PRISMA checklist. (DOCX)
